# Development of FRET and Stress Granule Dual-Based System to Screen for Viral 3C Protease Inhibitors

**DOI:** 10.3390/molecules28073020

**Published:** 2023-03-28

**Authors:** Jingjing Zhang, Yingpei Jiang, Chunxiu Wu, Dan Zhou, Jufang Gong, Tiejun Zhao, Zhigang Jin

**Affiliations:** 1 College of Life Sciences, Zhejiang Normal University, Jinhua 321004, China; 2 Key Laboratory of Novel Targets and Drug Study for Neural Repair of Zhejiang Province, School of Medicine, Hangzhou City University, Hangzhou 310015, China

**Keywords:** 3C protease, protease inhibitor, fluorescence resonance energy transfer, G3BP1, stress granules, Telaprevir, Trifluridine

## Abstract

3C proteases (3Cpros) of picornaviruses and 3C-like proteases (3CLpros) of coronaviruses and caliciviruses represent a group of structurally and functionally related viral proteases that play pleiotropic roles in supporting the viral life cycle and subverting host antiviral responses. The design and screening for 3C/3CLpro inhibitors may contribute to the development broad-spectrum antiviral therapeutics against viral diseases related to these three families. However, current screening strategies cannot simultaneously assess a compound’s cytotoxicity and its impact on enzymatic activity and protease-mediated physiological processes. The viral induction of stress granules (SGs) in host cells acts as an important antiviral stress response by blocking viral translation and stimulating the host immune response. Most of these viruses have evolved 3C/3CLpro-mediated cleavage of SG core protein G3BP1 to counteract SG formation and disrupt the host defense. Yet, there are no SG-based strategies screening for 3C/3CLpro inhibitors. Here, we developed a fluorescence resonance energy transfer (FRET) and SG dual-based system to screen for 3C/3CLpro inhibitors in living cells. We took advantage of FRET to evaluate the protease activity of poliovirus (PV) 3Cpro and live-monitor cellular SG dynamics to cross-verify its effect on the host antiviral response. Our drug screen uncovered a novel role of Telaprevir and Trifluridine as inhibitors of PV 3Cpro. Moreover, Telaprevir and Trifluridine also modulated 3Cpro-mediated physiological processes, including the cleavage of host proteins, inhibition of the innate immune response, and consequent facilitation of viral replication. Taken together, the FRET and SG dual-based system exhibits a promising potential in the screening for inhibitors of viral proteases that cleave G3BP1.

## 1. Introduction

Positive-sense single-stranded RNA (+ssRNA) viruses represent the largest group of RNA viruses, including picornaviruses, coronaviruses, and caliciviruses. Most of the pathogens in these viral families significantly impact on human and veterinary health. The classical and emerging human pathogens include poliovirus (PV), human enteroviruses (HEVs), human rhinoviruses (HRVs), Hepatitis A virus (HAV) and foot-and-mouth disease virus (FMDV) in picornaviruses, severe acute respiratory syndrome coronavirus (SARS-CoV), Middle East respiratory syndrome coronavirus (MERS-CoV) and SARS-CoV-2 in coronaviruses, and noroviruses and sapoviruses in caliciviruses [1,2,3,4]. During the replication of these +ssRNA viruses, one or more polyproteins translated directly from viral genome RNA are proteolytically cleaved into mature or intermediate viral proteins by viral proteases. Most of the cleavage tasks in picornaviruses, coronaviruses, and caliciviruses are assigned to 3C proteases (3Cpros) or 3C-like proteases (3CLpros). 3C/3CLpro share structural and functional similarities, including that they are 3-chymotrypsin-like cysteine proteases and contain a highly conserved three-dimensional structure with a Cys-His-Glu/Asp catalytic triad in 3Cpro or Cys-His dyad in 3CLpro and the preferential cleavage site Gln-Gly (P1–P1′). 3C/3CLpros are pleiotropic proteins other than those participating in the cleavage of viral polyproteins [3,4,5]. For example, picornaviral 3Cpro also possesses an RNA-binding activity and facilitates the assembly of the viral RNA replication complex and synthesis of viral RNA. In addition, picornaviral 3Cpro cleaves diverse host proteins, resulting in arrested host transcription and translation, and subverting antiviral host defenses. Recently, accumulating evidence has shown that 3C/3CLpro also play an important role in suppression of the host immune response by cleaving innate immune-related proteins, which is a critical viral strategy to support viral replication and pathogenesis [4,5,6].

By far, searching for 3C/3CLpro inhibitors has attracted much research attention to develop broad-spectrum antiviral therapeutics against picornaviruses, coronaviruses, and caliciviruses [2,3,5,7]. There are no homologs of 3C/3CLpro in humans, which further supports the strategy targeting 3C/3CLpro. A diversity of high-throughput screening and structure-based drug designs have been applied to discover 3C/3CLpro inhibitors, including library screening, laboratory synthesis, drug repositioning, and molecular docking [2,3,5,8,9,10]. These effects lead to a growing number of compounds identified as 3C/3CLpro inhibitors. According to the structure, they are mainly divided into peptides, heterocyclic esters, pyrazoles, isatin derivatives, and macrocyclics. 3C protease inhibitor Rupintrivir and its derivatives originally developed for HRV showed broad-spectrum antiviral activity against coronaviruses and other picornaviruses, indicating the potential of picornaviral 3Cpro inhibitors as broad-spectrum antiviral drugs [11,12]. However, only limited compounds have progressed to the clinical phase due to failure in infection conditions, biosafety, or other concerns. Thus, the development of antiviral 3C/3CLpro inhibitors is still ongoing.

Fluorescence resonance energy transfer (FRET) is a cell-free or cell-based approach frequently utilized in high-throughput drug screening [13,14,15,16]. After the SARS coronavirus outbreak in 2003, FRET has been used to evaluate the proteolytic activity of SARS-CoV 3CLpro and screen for its chemical inhibitors [17,18,19]. It was subsequently reported that FRET was applied to the antiviral drug screen in 3Cpro or 3CLpro-expressing viruses such as Coxsackievirus B3 (CVB3), HEV, noroviruses, and MERS-CoV [13,20,21,22]. Current structure- or protease-activity-based screening strategies are advantageous in compatibility with high throughput. However, they also have some limitations. For example, a single in vitro system is hardly able to evaluate a compound’s cytotoxicity and its impact on enzymatic activity as well as protease-mediated physiological processes simultaneously [23]. Therefore, the development of 3C/3CLpro inhibitors could still be improved by the innovation of new strategies or optimization of current strategies.

As we mentioned earlier, 3C/3CLpros are essential for the evasion of host immunity by cleaving innate immune-related proteins, such as RIG-I, MDA5, MAVS, NEMO, PKR, and G3BP1, most of which are associated with stress granules (SGs) [4,5,6]. SGs are cytoplasmic membraneless organelles assembled in response to environmental stress such as oxidative stress and virus invasion [24,25,26]. Upon viral infection, host cells deploy SGs as an important antiviral defense via blocking viral translation and stimulating the host immune response. G3BP1 is a key nucleation protein of SGs [27,28] and typically promotes antiviral immune signaling by recruitment of immune-related proteins to SGs for activation [29,30,31,32]. However, viruses have evolved various mechanisms to counteract SG formation, one of which is viral protease-mediated cleavage of G3BP1 [6,33,34]. Following viral dsRNA-triggered PKR activation, eIF2α phosphorylation, and induction of SGs in host cells, 3C/3CLpros prefer targeting G3BP1 for SG disassembly in later stages. For example, 3Cpro of PV, encephalomyocarditis virus (EMCV), enterovirus 71 (EV71), and CVB3 cleaves G3BP1 at the residue Q325, whereas FMDV 3Cpro cleaves G3BP1 at E285 [35,36,37,38,39]. In addition, 3CLpro of feline calicivirus (FCV) cleaves G3BP1 and disrupts the assembly of SGs [40]. A recent study showed that SARS-CoV-2 3CLpro (also known as Nsp5) disrupted SG formation, although it did not cleave G3BP1 [41]. Thus, G3BP1- and G3BP1-enriched SGs represent native substrates to reflect the protease activity of 3C/3CLpro in living cells. However, at present, there has been no attempt to establish an SG-based system to evaluate or screen for 3C/3CLpro inhibitors.

To conquer the above issues, here, we constructed a FRET-based biosensor of PV 3Cpro activity by coupling the N-terminal G3BP1 with cyan fluorescent protein (CFP) as the donor group and the C-terminal G3BP1 with yellow fluorescent protein (YFP) as the acceptor group. For cross-verification, we also established the cell line stably expressing green fluorescent protein (GFP)-tagged G3BP1 to live-monitor SG assembly. Taking advantage of this FRET and SG dual-based system, we tried a screen for PV 3Cpro inhibitors from an FDA-approved antiviral drug library and identified two compounds as novel PV 3Cpro inhibitors: hepatitis C virus (HCV) NS3/4Apro inhibitor Telaprevir and herpes simplex virus (HSV) replication inhibitor Trifluridine. Telaprevir and Trifluridine not only relieve the cleavage of G3BP1 and disruption of SGs by PV 3Cpro, but also restore the innate immune response inhibited by PV 3Cpro and viral replication facilitated by PV 3Cpro.

## 2. Results

### 2.1. G3BP1 Is Specifically Targeted by PV 3Cpro for Cleavage

**Figure 1:**
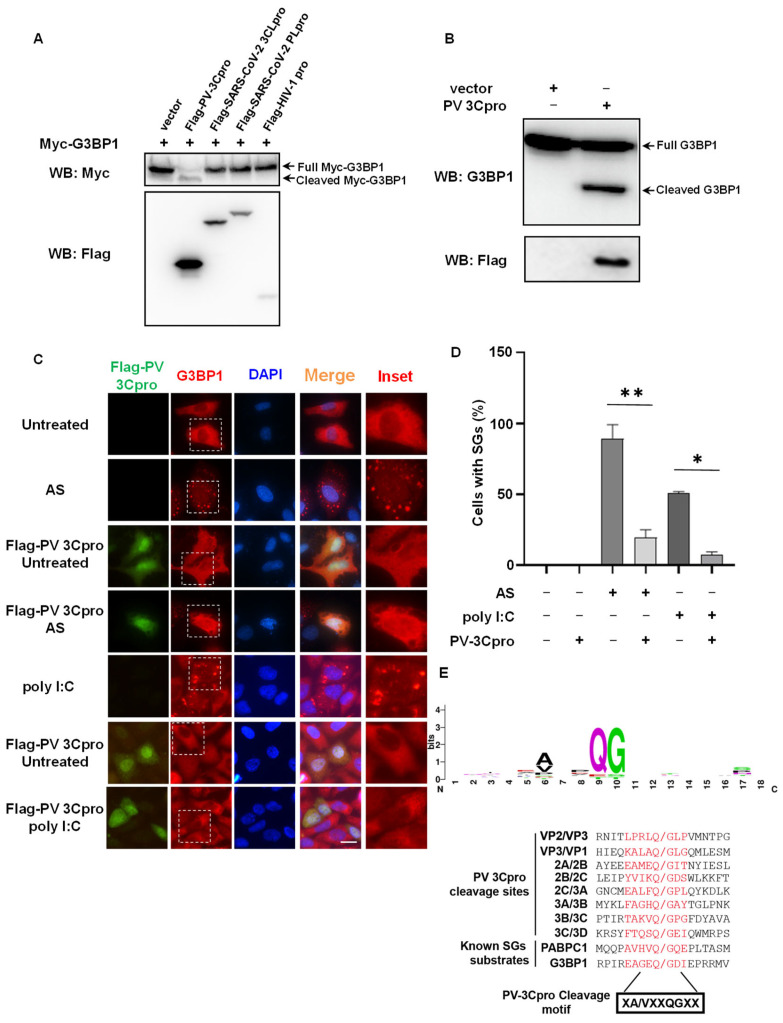
Cleavage of SG core protein G3BP1 by PV 3Cpro. (**A**) 293T cells were transfected with Flag-tagged viral proteases (PV 3Cpro, SARS-CoV-2 3CLpro and PLpro, HIV-1 pro) and Myc-G3BP1, followed by Western blot with anti-Flag and anti-Myc antibodies. (**B**) 293T cells were transfected with Flag-PV 3Cpro, followed by Western blot with anti-Flag and anti-G3BP1 antibodies. (**C**) HeLa cells were transfected with Flag-PV 3Cpro, and were untreated or treated with polyI:C for 9 h or 0.5 mM AS for 45 min followed by immunostaining for Flag (green) and G3BP1 (red). Scale bars: 20 μm. (**D**) Statistical analysis of the percentage of cells with SGs shown in panel (**C**). Data are shown as the mean ± SD (*n* = 3). Statistics: Student’s *t*-test (*, *p* < 0.05, **, *p* < 0.01). (**E**) Sequence logo of PV 3Cpro cleavage sites generated from 8 sites of PV polyproteins and 2 sites of host proteins. Amino acids are color-coded according to their physicochemical characteristics. Polar, green; basic, blue; neutral, purple; acidic, red; hydrophobic, black. Amino acids are shown as one-letter standard code. Cleavage nomenclature is according to Berger and Schechter.

In addition to viral polyproteins, 3C/3CLpros also target host proteins for cleavage, thereby subverting host defensive responses, among which G3BP1 is a preferential substrate of 3C/3CLpro [5,6]. To determine which types of viral proteases prefer SG core protein G3BP1 for cleavage, we co-transfected G3BP1 with various viral proteases in 293T cells, including PV 3Cpro, SARS2-CoV-2 3CLpro, and papain-like protease (PLpro) and HIV-1 protease. We found that only PV 3Cpro was able to cleave overexpressed Myc-G3BP1 (Figure 1A). Consistently, endogenous G3BP1 was also cleaved by PV 3Cpro (Figure 1B). Despite the infection of SARS-CoV-2 leading to the disruption of SG assembly [41,42,43,44,45], we found that SARS-CoV-2 3CLpro or PLpro did not cleave G3BP1 (Figure 1A). As G3BP1 is essential for SG assembly, we next investigated the effect of PV 3Cpro on SG assembly in PV 3C-expressing cells. Compared to the control groups with efficient induction of SGs by sodium arsenite (AS) or synthetic RNA duplex polyinosinic:polycytidylic acid (polyI:C), which is a mimic of RNA virus infection [41,46], the expression of PV 3Cpro significantly impaired the assembly of SGs (Figure 1C-D). _321_EAGEQGDI_328_, the cleavage site (P5–P3′) of G3BP1, is in perfect agreement with the consensus cleavage sequence of PV 3Cpro that we summarized from PV polyproteins and known host substrates (Figure 1E). These data indicate that the PV 3Cpro mediates the cleavage of G3BP1 and disruption of SG formation, which could be utilized to develop a G3BP1 and SG-based system to evaluate PV 3Cpro activity.

### 2.2. Establishment of FRET and SG Dual-Based System to Monitor PV 3Cpro Activity in Living Cells

**Figure 2:**
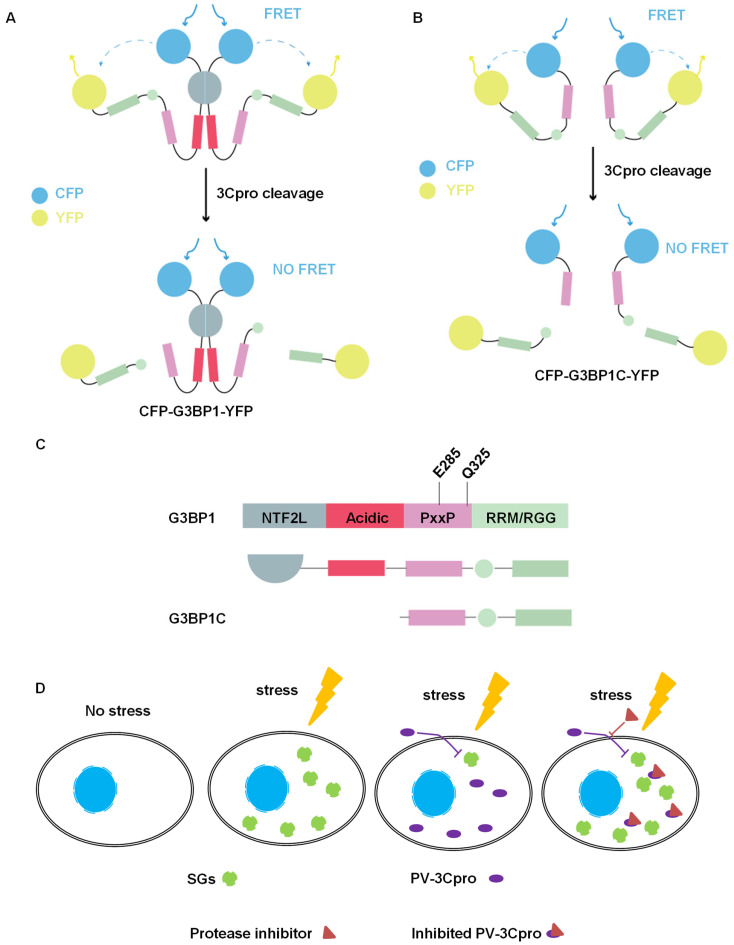
Schematic representation of FRET and SG dual-based system used for screening PV 3Cpro inhibitors. (**A**,**B**) Schematic graph showing the mechanism of FRET-based screening to monitor cleavage of G3BP1 by 3Cpro. (**C**) The cleavage sites of G3BP1 protein by picornaviral 3Cpro. Both the full-length and its C-terminal fragment were used to generate CFP-G3BP1-YFP and CFP-G3BP1C-YFP as substrates of PV 3Cpro. (**D**) Schematic graph showing the mechanism of SG-based screening to monitor SG dynamics.

In order to design a G3BP1-based biosensor as a detectable substrate of PV 3Cpro to reflect protease activity, we constructed a fusion plasmid CFP-G3BP1-YFP by tagging CFP and YFP, the donor and receptor pairs, to the N- terminal and C-terminal of G3BP1, respectively (Figure 2A). We reason that the close association of CFP and YFP will generate a FRET signal in cells transfected with CFP-G3BP1-YFP only. In contrast, in cells co-transfected with CFP-G3BP1-YFP and PV 3Cpro, the active PV 3Cpro will lead to cleavage of G3BP1, followed by the separation of CFP and YFP and loss of FRET signal. However, the inactivation of PV 3Cpro by a potential 3Cpro inhibitor would restore the FRET signal to a varying extent. In the case where the spatial distance between CFP and YFP in the context of full-length G3BP1 (466 amino acids) might impede the production of a strong FRET signal, we also used a C-terminal segment of G3BP1 (220–466, 247 amino acids), which is much shorter than full-length G3BP1 but retains the intact 3Cpro cleavage sites, and included the resultant CFP-G3BP1C-YFP into the FRET assay (Figure 2B,C). Viral protease-mediated cleavage of G3BP1 leads to its loss of function as well as a dominant negative effect [39]. Consequently, SGs are disassembled. Therefore, we also utilized HeLa cells stably expressing GFP-G3BP1 or GFP-G3BP2 to live-monitor SG dynamics [24] as a physiological outcome of PV 3Cpro activity (Figure 2D). In addition, the density, intensity, and subcellular localization of GFP in living cells could reflect the potential cytotoxicity of compounds.

**Figure 3:**
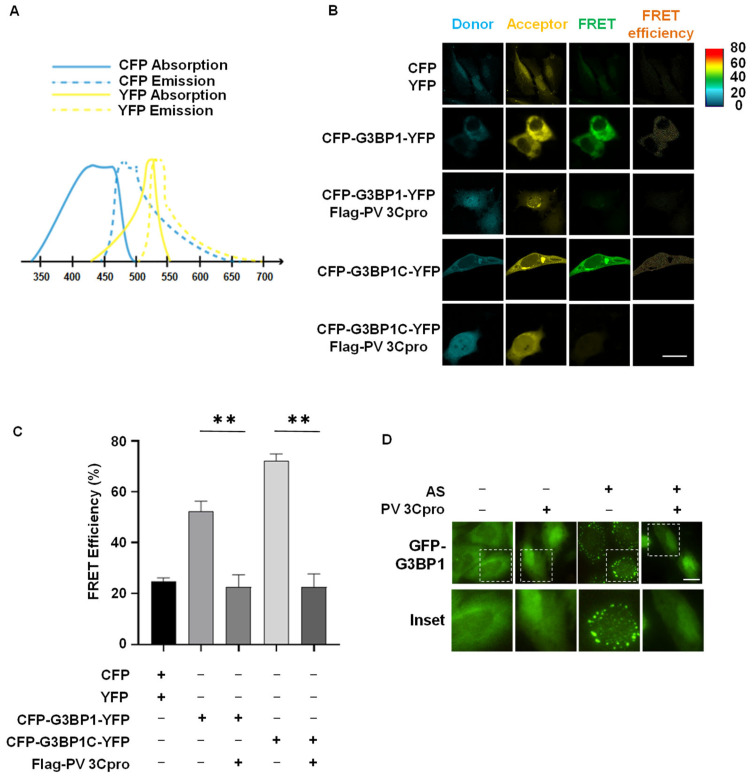
Monitoring PV 3Cpro activity in living cells via FRET and SG dual-based system. (**A**) Absorption spectrum of CFP (blue solid line) and emission spectra of CFP (blue dotted line), and absorption spectrum of YFP (yellow solid line) and emission spectra of YFP (yellow dotted line). (**B**) HeLa cells were transfected with CFP-G3BP1-YFP or CFP-G3BP1C-YFP together with Flag-PV 3Cpro or vectors followed by FRET using the FRET module of the Zeiss LSM880 confocal microscopy system. Scale bar: 20 μm. (**C**) Statistical analysis of the FRET efficiency shown in panel (**B**). Data are shown as the mean ± SD (*n =* 3). Statistics: Student’s *t*-test (**, *p* < 0.01). (**D**) HeLa cells stably overexpressing GFP-G3BP1 were transfected with Flag-PV 3Cpro or vectors, and were untreated or treated with 0.5 mM AS for 45 min followed by imaging of living cells. Scale bars: 20 μm.

As a next step, we tried to verify whether our FRET and SG dual-based system could assess PV 3Cpro activity in human cells. We transfected CYP-G3BP1-YFP or CYP-G3BP1C-YFP with or without PV 3Cpro into HeLa cells. A period of 36 h after transfection, we used a laser confocal microscope to detect the FRET acceptor signal at the wavelength of 514 nm after CFP was excited at 458 nm (Figure 3A). FRET efficiency was measured via the sensitized emission method and quantified by the Zeiss FRET Xia macro program. We found that HeLa cells transfected either with CYP-G3BP1-YFP or CYP-G3BP1C-YFP produced a FRET signal in the absence of PV 3Cpro (Figure 3C, column 3), with a more robust FRET efficiency from CYP-G3BP1C-YFP than CYP-G3BP1-YFP (Figure 3C column 4 and Figure 3D). However, FRET signals from CYP-G3BP1-YFP and CYP-G3BP1C-YFP were significantly compromised by the co-transfection of PV 3Cpro (Figure 3C, row 2 vs. row 3, row 4 vs. row 5), indicating that PV 3Cpro activity is inversely correlated to FRET efficiency. Consistently with the immunostaining results in Figure 1C showing live images of HeLa cells stably expressing GFP-G3BP1 as an SG marker, AS-induced SGs were blocked by the expression of PV 3Cpro (Figure 3D, column 3 vs. column 4). Thus, we successfully developed a FRET and SG dual-based system that could be used to screen for PV 3Cpro inhibitors in living cells.

### 2.3. Drug Screen Identified Telaprevir and Trifluridine as PV 3Cpro Inhibitors

**Figure 4:**
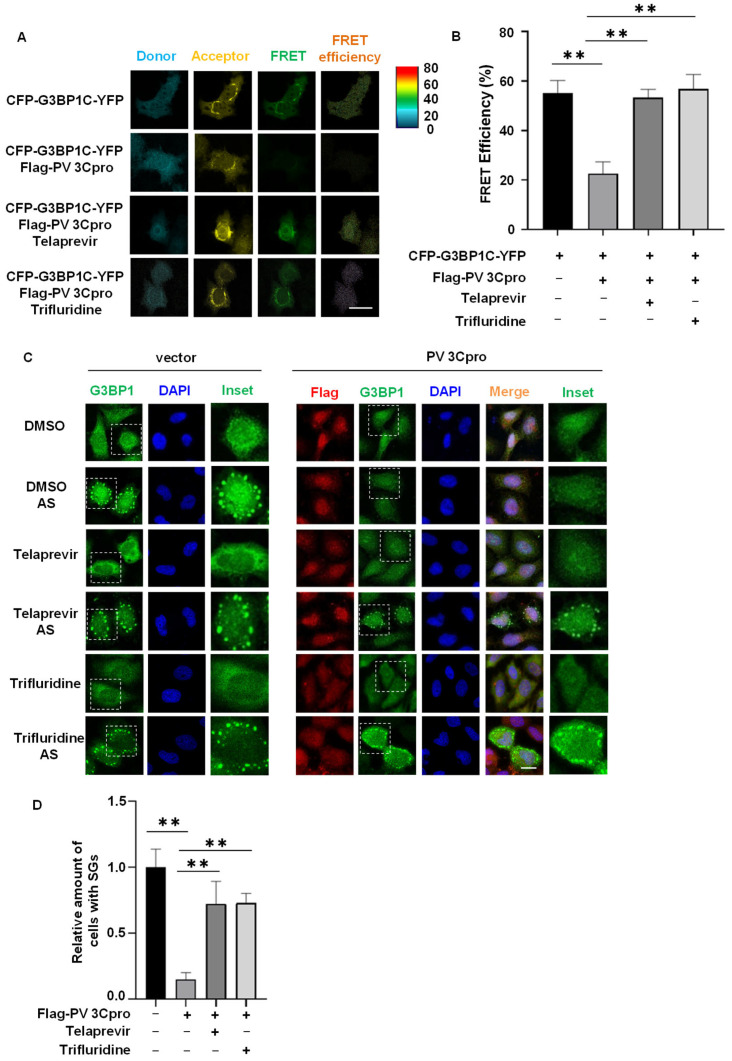
Identification of Telaprevir and Trifluridine as PV 3Cpro inhibitors by drug screening. (**A**) HeLa cells were transfected with CFP-G3BP1C-YFP together with Flag-PV 3Cpro or vectors, and were untreated or treated with 10 μM Telaprevir or Trifluridine 5 h after transfection, followed by FRET using the FRET module of the Zeiss LSM880 confocal microscopy system. Scale bar: 20 μm. (**B**) Statistical analysis of the FRET efficiency shown in panel (**A**). Data are shown as the mean ± SD (*n =* 3). Statistics: Student’s *t*-test (**, *p* < 0.01). (**C**) HeLa cells expressing GFP-G3BP1 were transfected with Flag-PV 3Cpro or vectors, and were untreated or treated with 10 μM Telaprevir or Trifluridine 5 h after transfection and incubated for 31 h. Cells were treated with 0.5 mM AS for 45 min followed by immunostaining for Flag (red). Scale bars: 20 μm. (**D**) Statistical analysis of the relative number of cells with SGs shown in panel (**C**). Data are shown as the mean ± SD (*n =* 3). Statistics: Student’s *t*-test (**, *p* < 0.01).

To verify the feasibility of our FRET and SG dual-based system for drug screening, we selected 64 known antiviral compounds from the FDA-approved drug library and carried out a screen for PV 3Cpro inhibitors (Table 1). Due to the higher FRET efficiency of CYP-G3BP1C-YFP than CYP-G3BP1-YFP, we transfected CYP-G3BP1C-YFP together with PV 3Cpro in HeLa cells and then treated with different compounds. Although the majority of compounds had little effect on either FRET or SG dynamics, Telaprevir and Trifluridine acted as inhibitors of PV 3Cpro by both the FRET screen and SG screen (Table 1). As shown in Figure 4A,B, the expression of PV 3Cpro reduced the FRET signal generated from CYP-G3BP1C-YFP, which could be rescued by treatment of Telaprevir or Trifluridine. It indicates that Telaprevir and Trifluridine are potential PV 3Cpro inhibitors. As the inhibition of PV 3Cpro-mediated cleavage of G3BP1 will result in the prevention of PV 3Cpro-mediated SG disassembly, we then investigated the effect of Telaprevir and Trifluridine on SGs in PV 3Cpro-expressing cells. Our results showed that the formation of SGs was significantly blocked by the expression of PV 3Cpro but restored by the treatment of Telaprevir or Trifluridine (Figure 4C,D). Thus, our screen repurposed Telaprevir and Trifluridine as a PV 3Cpro inhibitor. It also indicates the potential of the FRET and SG dual-based system in a high-throughput screen for inhibitors of PV 3Cpro as well as other 3C/3CLpros with the capacity of cleaving G3BP1.

### 2.4. Telaprevir and Trifluridine Counteract PV 3Cpro-Mediated Physiological Events

**Figure 5:**
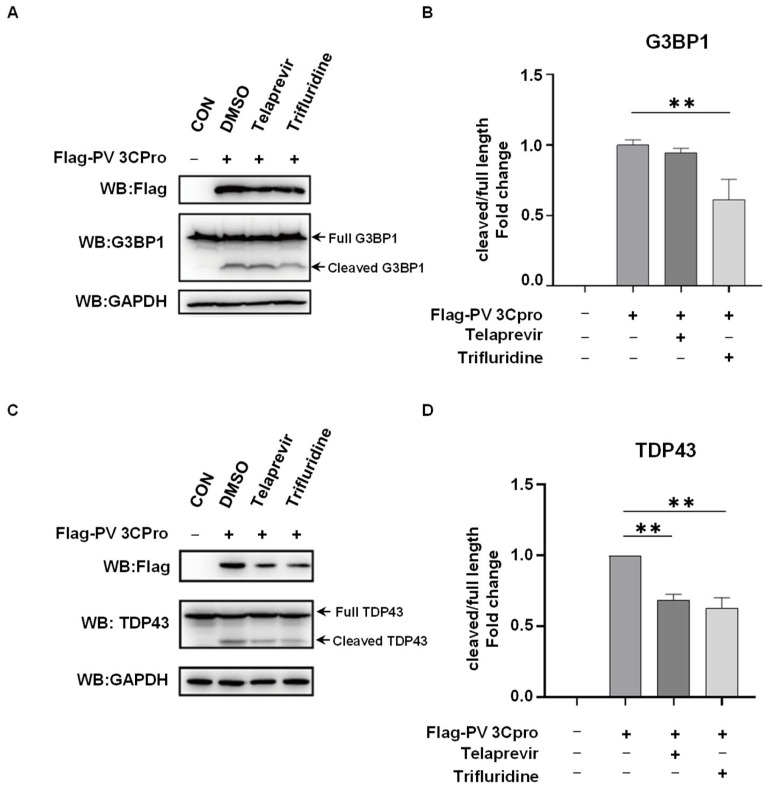
Telaprevir and Trifluridine suppress PV 3Cpro-mediated cleavage of host proteins. (**A**) 293T cells were transfected with Flag-PV 3Cpro or vectors, and were untreated or treated with 10 μM Telaprevir or Trifluridine 5 h after transfection, followed by Western blot with anti-Flag, anti-G3BP1, and anti-GAPDH antibodies. (**B**) Statistical analysis of the ratio of cleaved G3BP1 to full-length G3BP1 shown in panel (**A**). Data are shown as the mean ± SD (*n =* 3). Statistics: Student’s *t*-test (**, *p* < 0.01). (**C**) 293T cells were transfected with Flag-PV 3Cpro or vectors, and were untreated or treated with 10 μM Telaprevir or Trifluridine 5 h after transfection, followed by Western blot with anti-Flag, anti-TDP43, and anti-GAPDH antibodies. (**D**) Statistical analysis of the ratio of cleaved TDP43 to full-length TDP43 shown in panel (**C**). Data are shown as the mean ± SD (*n =* 3). Statistics: Student’s *t*-test (**, *p* < 0.01).

**Figure 6:**
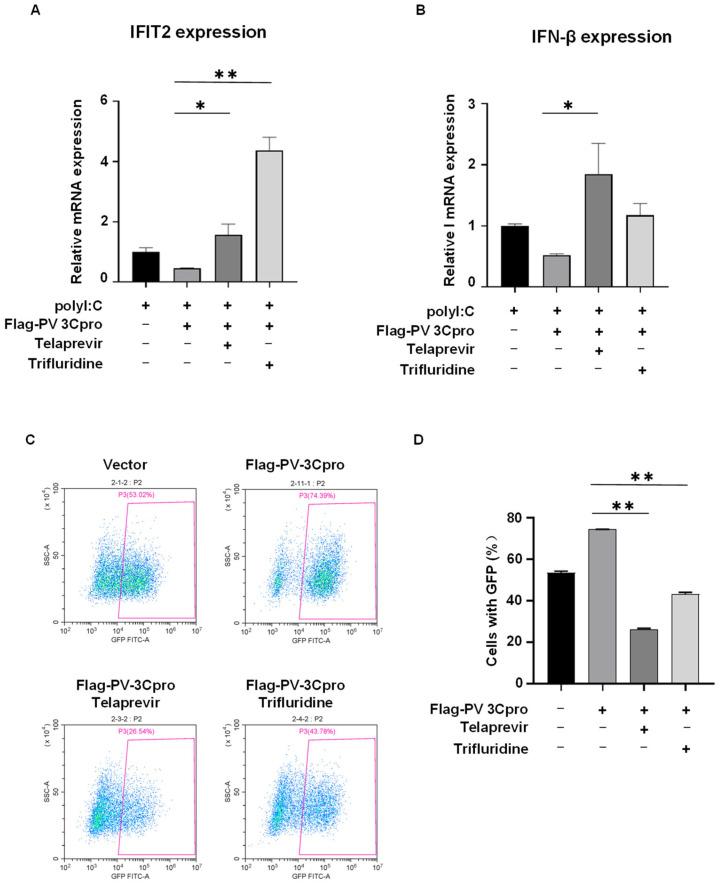
Telaprevir and Trifluridine counteract PV 3Cpro-mediated inhibition of innate immune response and facilitation of viral replication. (**A**,**B**) 293T cells were transfected with Flag-PV 3Cpro or vectors, and were untreated or treated with 10 μM Telaprevir and Trifluridine 5 h after transfection. A period of 22 h later, cells were transfected with polyI:C for 9 h and then harvested for RNA extraction and qPCR for IFIT2 (**A**) and IFN-β (**B**). Quantitative data are expressed as the means ± SD; *n =* 3. Statistics: Student’s *t*-test (*, *p* < 0.05, **, *p* < 0.01). (**C**) 293T cells were transfected with Flag-PV 3Cpro or vectors for 24 h, and infected by VSV-GFP, followed by treatment of 10 μM Telaprevir or Trifluridine for 12 h. Cells with GFP were measured by flow cytometric analysis. (**D**) Statistical analysis of the percentage of the cells with GFP shown in panel (**C**). Data are shown as the mean ± SD (*n =* 3). Statistics: Student’s *t*-test (**, *p* < 0.01).

PV 3Cpro has diverse physiological effects in host cells by the interaction with host proteins, such as the cleavage of host proteins, evasion of the immune response, and facilitation of viral replication [3,4,5]. We then investigated the impacts of Telaprevir and Trifluridine on PV 3Cpro-mediated physiological events. As shown in Figure 5, the expression of PV 3Cpro resulted in the cleavage of G3BP1 and TDP-43, both of which are known substrates of picornaviral 3Cpro [47,48]. This effect was attenuated by the treatment of Telaprevir or Trifluridine. In addition, PV 3Cpro blocked the polyI:C-induced mRNA expression of IFN-β and IFN-stimulated gene (ISG) IFIT2, which was restored by Telaprevir or Trifluridine (Figure 6A,B).

Finally, taking advantage of recombinant vesicular stomatitis virus (VSV) expressing GFP (VSV-GFP) as a model virus, we tried to determine whether the modulation of SGs and the innate immune response by PV 3Cpro and its inhibitors might play a role in regulating viral replication. HEK293T cells were transfected with PV 3Cpro followed by infection with VSV-GFP and compound treatment. We found that the expression of PV 3Cpro increased the percentage of GFP-positive cells compared to the control (Figure 6C,D). In contrast, Telaprevir and Trifluridine deprived PV 3Cpro of the supportive capacity, indicating that Telaprevir and Trifluridine reversed the PV 3Cpro-mediated facilitation of viral replication. Overall, Telaprevir and Trifluridine, obtained from our FRET and SG dual-based screen, counteract PV 3Cpro-mediated physiological events in host cells.

## 3. Discussion

3Cpro of picornaviruses and 3CLpro of coronaviruses and caliciviruses are a group of viral proteases with similar structure and function. They not only participate in the maturation of viral proteins, initiation of viral RNA synthesis, and other events in the viral life cycle, but also intervene in multiple cellular antiviral processes by cleaving host proteins. Not surprisingly, 3C/3CLpros have served as an attractive target for the development of antiviral drugs, especially broad-spectrum antiviral drugs against picornaviruses, coronaviruses, and caliciviruses. The efforts have led to the successful discovery of a series of 3C/3CLpro inhibitors, such as Rupintrivir (AG7088), AG7404, benserazide, dipeptidyl aldehyde (GC373), α-ketoamide (GC375), and dipeptidyl bisulfite adduct (GC376) [2,3,5,22,49]. To evaluate 3C/3CLpro inhibitors accurately, a system should mimic the scenario that occurs during viral infection in host cells as close as possible. Nevertheless, most of the current strategies for screening and evaluating 3C/3CLpro inhibitors are based on cell-free enzymatic assays with bacterially expressed 3C/3CLpro and a short peptide as the artificial substrate, thereby carrying with it some limitations [23]. For example, these assays neglect the effect of the post-translational modification of 3C/3CLpro that occurs in eukaryotic cells and the complicated protein structure of the substrate on the proteolytic reaction. In addition, they are unable to evaluate the cytotoxicity and physiological outcomes caused by the inhibition of 3C/3CLpro. To address these issues, here, we took advantage of G3BP1, a naïve substrate of 3C/3CLpro that is required for the assembly of antiviral SGs [35,36,37,38,39,40], and developed a FRET and SG dual-based system that could be used to evaluate and screen for 3C/3CLpro inhibitors in living cells. In this system, we expressed PV 3Cpro in human cells and co-expressed full-length (466 amino acids) or C-terminal (247 amino acids) G3BP1 to mimic PV 3Cpro-mediated cleavage of G3BP1 in PV-infected human cells. In addition to the evaluation of protease activity by FRET, our system also real-time-monitors SG dynamics so that the impact of protease activity on the antiviral stress response in host cells could be determined. Meanwhile, the cytotoxicity of a compound could also be reflected by the expression pattern of GFP. Thus, the FRET and SG dual-based system is able to simultaneously evaluate the protease activity as well as its physiological impact in living cells.

To verify the feasibility of the FRET and SG dual-based system, we performed a drug screen for PV 3Cpro inhibitors using an FDA-approved antiviral compound library. Although the novelty of our screen might be somewhat compromised by the limited number of well-documented compounds, drug repurposing from FDA-approved drugs could guarantee the biosafety of 3C/3CLpro inhibitors and accelerate the pace of drug development [50]. For example, a wide range of anti-HIV drugs have been successfully repurposed in cancer therapeutics and proposed for the treatment of COVID-19 recently [51,52]. Of note, we intended to include protease inhibitors for other viruses such as HCV and HIV into our compound library. Our screen identified two compounds that are able to inhibit PV 3Cpro activity, Telaprevir and Trifluridine. Telaprevir is a known HCV NS3/4Apro inhibitor approved by FDA for the treatment of HCV infection [53]. Trifluridine is a known HSV replication inhibitor and used as an antiviral drug for HSV infection [54]. Interestingly, both HCV and HSV infections induce or modulate the assembly of SGs [55,56,57,58]. It is interesting to know whether HCV NS3pro is also involved in the cleavage of G3BP1, disassembly of SGs, and suppression of host immunity during HCV infection. It also indicates that Telaprevir might play a common role against PV and HCV by the inhibition of 3Cpro and NS3pro.

If Telaprevir and Trifluridine are bona fide PV 3Cpro inhibitors, they should also affect PV 3Cpro-mediated cellular events, such as the cleavage of host proteins and evasion of the host innate immune response. The modulation of these host responses by PV 3Cpro will finally provide a favorable environment for viral replication. We verified that Telaprevir and Trifluridine repressed the cleavage of G3BP1 by PV 3Cpro. Accordingly, Telaprevir and Trifluridine relieved the inhibitory effect of PV 3Cpro on the innate immune response. Finally, the facilitation of viral replication by PV 3Cpro was overridden by Telaprevir and Trifluridine. Thus, we were able to verify the physiological outcomes following inhibition of PV 3Cpro by Telaprevir and Trifluridine. However, whether Telaprevir and Trifluridine inhibit PV 3Cpro and viral replication in the context of PV infection need further investigation.

During the screening using the FRET and SG dual system, we noticed that five compounds were positive in the FRET assay but negative in the SG assay. We believed that this is due to the false positive that is inevitable in a single fluorescence-intensity-based assay, such as FRET. In the FRET assay, the false positive results are usually caused by chemical interferences, such as dyes and aggregators [59,60]. To avoid this problem, we introduced an SG-based assay to verify the physiological outcome of FRET hits. We believe the FRET and SG dual-based system will reduce the false positive rate compared to a single system.

Our method still has certain limitations. Our FRET-based system could be applied to a range of viral proteases that cleave G3BP1 but not to other viral proteases that do not cleave G3BP1. Different viral proteases usually recognize consensus sites with specific sequences to fulfill the substrate specificity. As most of the 3C proteases could cleave G3BP1, we believe our FRET-based system could be applied to screen for 3C protease inhibitors. To screen for inhibitors of other viral proteases, their cleavage sites derived from either viral polyproteins or host substrates could be used to replace G3BP1 and construct a similar FRET-based system. We also tried to include a known PV 3Cpro inhibitor as a positive control but failed due to commercial unavailability. We only used PV 3Cpro for evaluation, and only determined the drug treatment time according to the reported period when PV 3Cpro disassembled SGs. The system could be improved in several ways, such as optimization of the treatment time and concentration of the compound. In addition, although our current FRET system is suitable for high-throughput screening, the SG system is not yet suitable, as we manually counted the GFP-G3BP1-positive SGs and calculated the percentage of cells with SGs. However, this could be improved in several ways, for example, by utilizing the granule-counting software AggreCount [61] or high-content imaging system. We believe that the FRET and SG dual-based system would be available for high-throughput screening in the future.

## 4. Materials and Methods

### 4.1. Cell Culture and Transfection

Human embryonic kidney epithelial cell line (293T) and human cervical cancer cells (HeLa) were grown in Dulbecco’s modified Eagle’s medium (DMEM, BasalMedia, Shanghai, China) supplemented with 10% fetal bovine serum (ExCell Bio, Shanghai, China) and 1% penicillin/streptomycin (Gibco, Gaithersburg, MD, USA) under standard tissue-culture conditions (37 °C, 5% CO_2_). HeLa cells stably overexpressing GFP-G3BP1 or GFP-G3BP2 were grown in DMEM with 10% FBS and 2 μg/mL of puromycin [24]. The cells were seeded in multiwell plates or dishes and transfected with plasmids at approximately 60~70% confluence using PEI (Polyscience, Niles, IL, USA) or lipofectamine 2000 (ThermoFisher, Waltham, MA, USA).

### 4.2. Plasmids and Reagents

Viral genes encoding viral proteases were synthesized and cloned into vector pCS2-Flag (Tsingke, Beijing, China). CFP and YFP cDNA were subcloned into the pCS2 vector by PCR amplification to generate pCS2-CFP-YFP. Full-length human G3BP1 or its C-terminal fragment (220–466, 247 amino acids) was subcloned to sites between CFP and YFP in pCS2-CFP-YFP to prepare pCS2-CFP-G3BP1-YFP or pCS2-CFP-G3BP1C-YFP. A total of 64 antiviral compounds were selected from the DiscoveryProbe™ FDA-approved drug library (APExBIO #L1021, Shanghai, China). The following antibodies were used in Western blot and Immunofluorescence: Flag (Sigma, St. Louis, MO, USA, #F1804 and #F7425), Myc (Sigma, St. Louis, MO, USA, #M5546), G3BP1 (Santa Cruz Biotech, Dallas, TX, USA, #81940), TDP-43 (ProteinTech, Wuhan, China, #10782-2-AP), and GAPDH (ProteinTech, Wuhan, China, # 60004-1-Ig).

### 4.3. SG Induction and Quantification

PolyI:C (Sigma, St. Louis, MO, USA) was dissolved in RNase-free water containing 0.98% NaCl to make 5 mg/mL of stock solution. Before use, polyI:C was incubated at 50 °C for 20 min followed by slow cooling to room temperature for annealing. To mimic stress induced by the viral replication of intermediate dsRNA, 1 μg/mL of polyI:C was transfected into the cells with lipofectamine 2000 (ThermoFisher, Waltham, MA, USA) for 9 h or the indicated time. To induce oxidative stress, the cells were treated with 0.5 mM AS (Sigma, St. Louis, MO, USA) for 45 min. G3BP1 was used as a marker of polyI:C or AS-induced SGs. For SG counting, the percentage of cells with SGs was analyzed in 50 cells per condition and a minimum of three granules per cell were required to score as positive.

### 4.4. Immunofluorescence

Cells were fixed in 4% paraformaldehyde (PFA) for 30 min at 4 °C and washed with PBS three times. Next, the fixed cells were incubated in blocking solution containing 1% BSA (Sigma, St. Louis, MO, USA) and 1% normal goat serum (Jackson, West Grove, PA, USA) in PBS with 0.1% Triton X-100 for 30 min at room temperature. Cells were then incubated with primary antibodies overnight at 4 °C. The next day, the cells were washed three times with PBS and incubated with secondary antibodies conjugated to Alexa Fluor 488 (ThermoFisher, Waltham, MA, USA) or Alexa Fluor 594 (ThermoFisher, Waltham, MA, USA) for 1 h at room temperature. Cell nuclei were stained with DAPI (Sigma, St. Louis, MO, USA). Images were acquired with a Zeiss LSM880 microscope (Oberkochen, Germany).

### 4.5. Protein Extraction and Western Blotting

Protein extraction and Western blotting were performed as described previously [43]. 293T cells were seeded to reach 60~70% confluence in 6-well plates or 12-well plates, and transfected with plasmids for 36 h. After treatment, the cells were washed with PBS and lysed in lysis buffer containing 50 mM Tris HCl, pH 7.6, 150 mM NaCl, 0.5% NP40, 1 mM EDTA, and a protease inhibitor at 4 °C for 10 min. Cell lysates were centrifuged twice for 5 min (14,000 rpm, 4 °C), and the supernatants were used for Western blotting after boiling in SDS loading buffer for 5 min, 95 °C.

### 4.6. The Fluorescence Resonance Energy Transfer (FRET) Assay

The FRET assay was performed using the FRET module of the Zeiss LSM880 confocal microscopy system and FRET efficiency was evaluated by the sensitized emission method [62]. HeLa cells were seeded to reach 60~70% confluence in 6-well plates with coverslips and transfected with plasmids. After transfecting for 5 h, the cells were treated with 10 μM of compound for 31 h. Images were acquired with the Zeiss LSM880 microscope with the following channels: donor-CFP (column 1, excitation at 458 nm and emission at 463–520 nm), acceptor-YFP (column 2, excitation at 514 nm and emission at 520–620 nm), and FRET (column 3, excitation at 458 nm and emission at 520–620 nm). Images sequentially obtained from the CFP, YFP, and FRET channels were processed using a Zeiss FRET Xia macro program, in which FRET signals were subtracted by bleed-through from CFP and YFP channels to obtain net FRET (NFRET) and compute the FRET efficiency (E, column 4). For quantification of the FRET efficiency, 1–2 cells in the same field of view and a total of 3 fields were analyzed per condition and repeated in triplicate experiments.

### 4.7. RNA Extraction, Reverse Transcription, and Quantitative PCR 

To detect the polyI:C-induced innate immune response, 293T cells in 6-well plates were transfected with plasmid pCS2-Flag-PV 3Cpro or vectors. A period of 5 h later, cells were treated with 10 μM of compound or DMSO for 22 h followed by transfection of 1 μg/mL of polyI:C for an additional 9 h. Total RNA was then isolated from 293T cells by utilizing RNAiso Plus reagent (Takara, Japan) according to the manufacturer’s instructions. An amount of 1 μg of total RNA was used for reverse transcription with the Prime Script™ RT Reagent kit with gDNA Eraser (Takara, Japan). Quantitative PCR was performed in triplicate using SYBR green master mix (ThermoFisher, Waltham, MA, USA) on an Applied Biosystems (South San Francisco, CA, USA) 7500 Real-Time PCR System. The expression level of mRNAs was normalized to that of β-actin. Primers for the following human genes were used: β-actin (forward: 5′-AGCGGGAAATCGTGCGTGAC-3′; reverse: 5′-CAATGGTGATGACCTGGCCGT-3′), IFN-β (forward: 5′-ATGACCAACAAGTGTCTCCTCC-3′; reverse: 5′-GGAATCCAAGCAAGTTGTAGCTC-3′), and IFIT2 (forward: 5′-AAGCACCTCAAAGGGCAAAAC-3′, reverse: 5′-TCGGCCCATGTGATAGTAGAC-3′).

### 4.8. Viral Infection and Flow Cytometry Analysis

GFP-labeled VSV was used to infect 293T cells as described previously [63]. Briefly, 293T cells were transfected with plasmid pCS2-Flag-PV 3Cpro for 24 h. VSV-GFP was then diluted to the MOI of 1:100 with serum-free DMEM medium and infected 293T cells for 2 h. Infected 293T cells were then refreshed by DMEM containing 10% FBS and treated with 10 μM of compound or DMSO. After 12 h, infected 293T cells were harvested by trypsinization for flow cytometry to detect the positive rate of GFP cells (no less than 10,000 cells in each group).

### 4.9. Statistical Analyses

Statistical analyses were performed using GraphPad Prism software. All results are expressed as means ± SD of at least three independent experiments (*n* ≥ 3). The *p* value was calculated using an unpaired Student’s *t*-test. Significance was assumed for * *p* < 0.05 and ** *p* < 0.01. The sequence logo of PV 3Cpro cleavage sites was analyzed by WebLogo (http://weblogo.berkeley.edu/, accessed on 8 January 2022) [64].

## 5. Conclusions

In summary, our study established a FRET and SG dual-based system to evaluate and screen for 3C/3CLpro inhibitors in living cells. Utilizing this system, our screen uncovered a novel role of HCV NS3/4Apro inhibitor Telaprevir and HSV replication inhibitor Trifluridine as the PV 3Cpro inhibitor. Telaprevir and Trifluridine are able to counteract PV 3Cpro-mediated SG disassembly, the cleavage of host proteins, and the modulation of the host immune response and viral replication. Although we only used this system to screen for PV 3Cpro inhibitors in this study, our system could also be applied to screen for inhibitors of other 3C/3CLpro that target G3BP1 for cleavage, such as 3Cpro of EMCV, EV71, FMDV, and CVB3 and 3CLpro of FCV [35,36,37,38,39,40].

## Figures and Tables

**Table 1:** FDA-approved antiviral compound used in this study and summary of the screening results.

Number	Name	CAS	Description	FRET Screen	SGs Screen
1	Amprenavir	161814-49-9	HIV protease inhibitor	−	−
2	Ritonavir	155213-67-5	HIV protease inhibitor	−	−
3	Lopinavir	192725-17-0	HIV protease inhibitor	−	−
4	Atazanavir	198904-31-3	HIV protease inhibitor	+	−
5	Darunavir	206361-99-1	HIV protease inhibitor	−	−
6	Saquinavir mesylate	149845-06-7	HIV protease inhibitor	−	−
7	Nelfinavir	159989-64-7	HIV protease inhibitor	−	−
8	Asunaprevir	630420-16-5	HCV NS3pro inhibitor	+	−
9	Boceprevir	394730-60-0	HCV NS3pro inhibitor	−	−
10	Ledipasvir	1256388-51-8	HCV NS5Apro inhibitor	−	−
11	Simeprevir	923604-59-5	HCV NS3/4Apro inhibitor	−	−
12	Telaprevir	402957-28-2	HCV NS3/4Apro inhibitor	+	+
13	Daclatasvir	1009119-64-5	HCV NS5Apro inhibitor	−	−
14	Tenofovir Disoproxil Fumarate	202138-50-9	HIV-1 reverse transcriptase (RT) inhibitor	−	−
15	Entecavir Hydrate	209216-23-9	HBV replication inhibitor	−	−
16	Abacavir	136470-78-5	HIV RT inhibitor	−	−
17	MK-5172	1350514-68-9	HCV NS3/4A pro inhibitor	−	−
18	Oseltamivir	196618-13-0	Influenza neuraminidase inhibitor	−	−
19	Oseltamivir acid	187227-45-8	Influenza neuraminidase inhibitor	−	−
20	Peramivir	330600-85-6	Influenza neuraminidase inhibitor	+	−
21	PSI-7977	1190307-88-0	HCV NS5B polymerase inhibitor	−	−
22	Rilpivirine	500287-72-9	nonnucleoside RT inhibitor	−	−
23	Lomibuvir (VX-222)	1026785-59-0	HCV NS5B polymerase inhibitor	−	−
24	Elvitegravir	697761-98-1	HIV-1 integrase inhibitor	−	−
25	Raltegravir	518048-05-0	HIV-1 integrase inhibitor	+	−
26	S/GSK1349572	1051375-16-6	HIV-1 integrase inhibitor	−	−
27	Fumagillin	23110-15-8	Methionine aminopeptidase-2inhibitor	−	−
28	Tenofovir	147127-20-6	HIV RT inhibitor	−	−
29	Cidofovir	113852-37-2	viral DNA synthesis inhibitor	−	−
30	Maraviroc	376348-65-1	CCR5 inhibitor	−	−
31	Arbidol HCl	131707-23-8	viral fusion inhibitor	−	−
32	Didanosine	69655-05-6	RT inhibitor	−	−
33	Emtricitabine	143491-57-0	RT inhibitor	−	−
34	Lamivudine	134678-17-4	Nucleoside analog RT inhibitor	−	−
35	Nevirapine	129618-40-2	Non-nucleoside RT inhibitor	−	−
36	Trifluridine	70-00-8	HSV replication inhibitor	+	+
37	Acyclovir	59277-89-3	viral replication inhibitor	−	−
38	Favipiravir (T 705)	259793-96-9	RNA-dependent RNA polymerase inhibitor	−	−
39	Efavirenz	154598-52-4	RT inhibitor	+	−
40	Idoxuridine	54-42-2	nucleoside analogues	−	−
41	Oseltamivir phosphate	204255-11-8	Neuraminidase inhibitor	+	−
42	Penciclovir	39809-25-1	neuraminidase inhibitor	−	−
43	Salicylanilide	87-17-2	antiviral	−	−
44	Valganciclovir	175865-59-5	viral DNA polymerase inhibitor	−	−
45	Famciclovir	104227-87-4	Hsv-2 polymerase inhibitor	−	−
46	Moroxydine HCl	3160-91-6	Virus Proliferation inhibitor	−	−
47	Valaciclovir	124832-27-5	virus DNA polymerase inhibitor	−	−
48	Vidarabine	5536-17-4	viral DNA synthesis inhibitor	−	−
49	Ganciclovir	82410-32-0	Viral replication inhibitor	−	−
50	Ribavirin	36791-04-5	antiviral	−	−
51	Zanamivir	139110-80-8	Influenza A/B virus neuraminidases inhibitor	−	−
52	Peramivir Trihydrate	1041434-82-5	Influenza viral neuraminidase inhibitor	−	−
53	Abacavir sulfate	188062-50-2	RT inhibitor	−	−
54	Adefovir Dipivoxil	142340-99-6	RT inhibitor	−	−
55	Zalcitabine	7481-89-2	RT inhibitor	−	−
56	Etravirine (TMC125)	269055-15-4	RT inhibitor	−	−
57	Stavudine (d4T)	3056-17-5	RT inhibitor	−	−
58	GSK1349572	1051375-19-9	HIV integrase inhibitor	−	−
59	Rimantadine	1501-84-4	M2 proton channel inhibitor	−	−
60	GS-7340	379270-37-8	HIV RT inhibitor	−	−
61	Rolipram	61413-54-5	PDE4 selective inhibitor	−	−
62	Telbivudine	3424-98-4	RT inhibitor	−	−
63	Artemisinine	63968-64-9	AKT signaling pathway inhibitor	−	−
64	Cepharanthine	481-49-2	viral proliferation inhibitor	−	−

## Data Availability

All generated and analyzed data used to support the findings of this study are included within the article.

## Sample Availability

Samples of the compounds are available from the authors.
